# Imaging topology of Hofstadter ribbons

**Published:** 2019

**Authors:** Dina Genkina, Lauren M Aycock, Hsin-I Lu, Mingwu Lu, Alina M Pineiro, I B Spielman

**Affiliations:** 1 Joint Quantum Institute, National Institute of Standards and Technology, and University of Maryland, Gaithersburg, MD 20899, United States of America; 2 Currently Modern Electron, Bellevue, WA 98007, United States of America

**Keywords:** ultracold atoms, quantum simulation, quantum Hall effect, quantum transport

## Abstract

Physical systems with non-trivial topological order find direct applications in metrology (Klitzing *et al* 1980 *Phys. Rev. Lett.* **45** 494–7) and promise future applications in quantum computing (Freedman 2001 *Found. Comput. Math.* **1** 183–204; Kitaev 2003 *Ann. Phys.* **303** 2–30). The quantum Hall effect derives from transverse conductance, quantized to unprecedented precision in accordance with the system’s topology (Laughlin 1981 *Phys. Rev.* B **23** 5632–33). At magnetic fields beyond the reach of current condensed matter experiment, around 10^4^ T, this conductance remains precisely quantized with values based on the topological order (Thouless *et al* 1982 *Phys. Rev. Lett.* **49** 405–8). Hitherto, quantized conductance has only been measured in extended 2D systems. Here, we experimentally studied narrow 2D ribbons, just 3 or 5 sites wide along one direction, using ultracold neutral atoms where such large magnetic fields can be engineered (Jaksch and Zoller 2003 *New J. Phys.* **5** 56; Miyake *et al* 2013 *Phys. Rev. Lett.* **111** 185302; Aidelsburger *et al* 2013 *Phys. Rev. Lett.* **111** 185301; Celi *et al* 2014 *Phys. Rev. Lett.* **112** 043001; Stuhl *etal* 2015 *Science* **349** 1514; Mancini *et al* 2015 *Science* **349** 1510; An *et al* 2017 *Sci. Adv.* **3**). We microscopically imaged the transverse spatial motion underlying the quantized Hall effect. Our measurements identify the topological Chern numbers with typical uncertainty of 5%, and show that although band topology is only properly defined in infinite systems, its signatures are striking even in nearly vanishingly thin systems.

## 1. Introduction

The importance of topology in physical systems is famously evidenced by the quantum Hall effect’s role as an ultra-precise realization of the von Klitzing constant *R*_*K*_ = *h/e*^2^ of resistance [1]. Although topological order is only strictly defined for infinite systems, the bulk properties of macroscopic topological systems closely resemble those of the corresponding infinite system. For 2D systems in a magnetic field *B*_0_, the topology is characterized by an integer invariant called the Chern number. Even at laboratory fields of tens of Tesla, crystalline materials have a small magnetic flux Φ = *AB*_0_ per individual lattice plaquette (with area *A*) compared to the flux quantum Φ_0_ = *h/e*. Superlattice [2–5] and ultracold atom [6–9] systems now realize 2D lattices in a regime where the magnetic flux per plaquette Φ is a significant fraction of Φ_0_.

Experimental signatures of Chern numbers generally leverage one of two physical effects: in condensed matter systems the edge-bulk correspondence allows the Chern number to be inferred from the quantized Hall conductivity *σ*_*H*_ = *C/R*_*K*_, and in cold-atom experiments direct probes of the underlying band structure at every value of crystal momentum give access to the Chern number through either static [10,11] or dynamic [12–15] signatures. Both of these connections derive from the pioneering work of Thouless, Kohmoto, Nightingale, and denNijs [16], in the now famous TKNN paper. Going beyond these well known techniques, the TKNN paper showed that for rational flux Φ/Φ_0_ = *P/Q* (for relatively prime integers *P* and *Q*) the integer solutions *s* and *C* to the Diophantine equation

(1) 1=Qs−PC

uniquely^3^ determine the Chern number *C* of the lowest band. Following theoretical work [11,17–20], we leveraged this TKNN equation to determine the Chern number of our system.

## 2. Experimental setup

**Figure 1.**
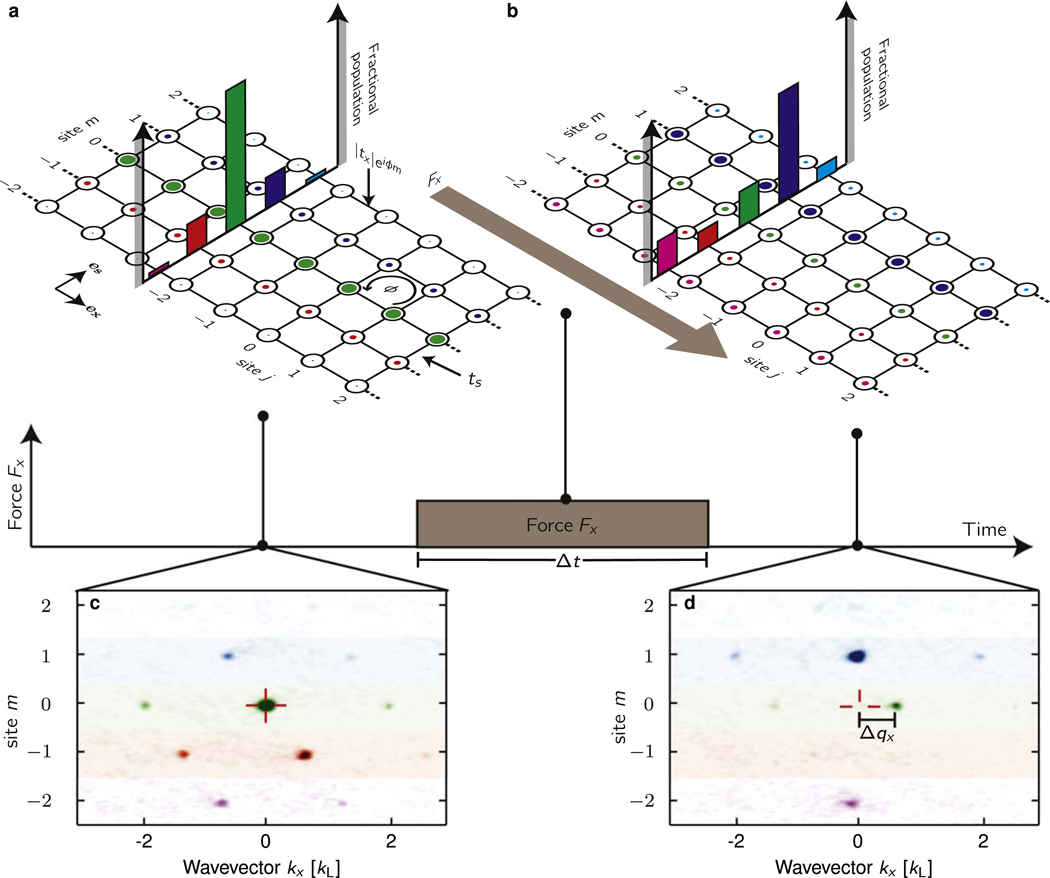
Quantum Hall effect in Hofstadter ribbons. (a) 5-site wide ribbon with real tunneling coefficients along **e**_*s*_ and complex tunneling coefficients along **e**_*x*_, creating a non-zero phase *ϕ* around each plaquette. (b) After applying a force along **e**_*x*_ for a time Δ*t*, atomic populations shift transverselyalong **e**_*s*_, signaling the Hall effect. (c), (d) TOF absorption images giving hybrid momentum/position density distributions *n*(*k*_*x*_, *m*). Prior to applying the force (c),the *m* = 0 momentum peak is at *k*_*x*_ = 0, marked by the red cross. Then, in (d), the force directly changed *q*_*x*_, evidenced by the displacement Δ*q*_*x*_ of crystal momentum, andvia the Hall effect shifted population along **e**_*s*_.

We studied ultracold neutral atoms in a square lattice with a large magnetic flux per plaquette. As pictured in figure 1(a), our system consisted of a 2D lattice that was extremely narrow along one direction, just 3 or 5 sites wide—out of reach of traditional condensed matter experiments, with hard wall boundary conditions: a ribbon. Our system was qualitatively well described by the Harper-Hofstadter Hamiltonian in the Landau gauge [24,25]

(2) $$\hat{H}=-\sum_{m,j}\left(t_{x}{\text{e}}^{\text{i}\phi m}|j,m\rangle \;\langle j+1,m\left|+t_{s}\right|j,m\rangle \;\langle j,m+1|\right)+\text{h}.\text{c}.,$$

where *j* and *m* label lattice sites along **e**_*x*_ and **e**_*s*_, with tunneling strengths *t*_*x*_ and *t*_*s*_ respectively. As shown in figure 1(a), tunneling along **e**_*x*_ was accompanied by a phase shift e^i*ϕm*^. Hopping around a single plaquette of this lattice imprints a phase *ϕ*, analogous to the Aharanov–Bohm phase, emulating a magnetic flux $\Phi /\Phi_{0}=\phi /2\pi .$ We implemented this 2D lattice by combining a 1D optical lattice defining sites along an extended direction **e**_*x*_, with atomic spin states forming lattice sites along a narrow, synthetic [26–28,29] direction **e**_*s*_. The exact Hamiltonian of the underlying atomic system differs from the Harper–Hofstadter Hamiltonian above in that *t*_*s*_ is non-uniform due to Clebsch–Gordan coefficients and there is a small *m*^2^ dependent potential term due to the quadratic Zeeman shift (see appendix D).

This system exhibits a Hall effect, where a longitudinal force $F_{\|}$—analogous to the electric force $eE_{\|}$ in electronic systems—drives a transverse ‘Hall’ current density $j_{⊥}=\sigma_{\text{H}}E_{\|}$ for non-zero Φ/Φ_0_. A longitudinal force *F*_*x*_ would drive a change in the dimensionless crystal momentum $\hbar \Delta q_{x}/\hbar G$ and a transverse displacement Δ*m*, giving a dimensionless Hall conductivity $NG\Delta m/\Delta q_{x}=\sigma_{\text{H}}R_{\text{K}}={\tilde{\sigma }}_{\text{H}},$ where *G* is the reciprocal lattice constant and *N* is the number of carriers per plaquette (see appendix C). Starting with Bose-condensed atoms in the lattice’s ground state (with transverse density shown in figure 1(a)) we applied a force along **e**_*x*_ and obtained Δ*m* from site resolved density distributions [11] along **e**_*s*_ (figure 1(b)). Leveraging the TKNN equation (equation (1)), we further show that the force required to move the atoms a single lattice site signals the infinite system’s Chern number.

Our quantum Hall ribbons were created with optically trapped ^87^Rb Bose–Einstein condensates (BECs) in either the *F* = 1 or 2 ground state hyperfine manifold, creating 3 or 5 site-wide ribbons from the 2*F* + 1 states available in either manifold. We first loaded BECs into a 1D optical lattice along **e**_*x*_ formed by a retro-reflected λ_L_ = 1064 nm laser beam. This created a lattice with period *a* = λ_L_/2 and depth 4.4(1) *E*_L_, giving tunneling strength *t*_*x*_ = 0.154(4)*E*_L_. Here, $E_{\text{L}}=\hbar^{2}k_{\text{L}}^{2}/2m_{\text{Rb}}$ is the single photon recoil energy; $\hbar k_{\text{L}}=2\pi \hbar /\lambda_{\text{L}}$ is the single photon recoil momentum; and *m*_Rb_ is the atomic mass. We induced tunneling along **e**_*s*_ with either a spatially uniform rf magnetic field or two-photon Raman transitions. The tunneling strength was *t*_*s*_ = 1.97(8) *t*_*x*_ for Raman coupling in the *F* = 2 manifold, *t*_*s*_ = 0.97(8) *t*_*x*_ for Raman coupling in the *F* = 1 manifold, and *t*_*s*_ = 7.4(5) *t*_*x*_ for rf coupling in both manifolds. The rf-induced tunneling imparted at most only a spatially uniform tunneling phase, giving *ϕ*/2*π* = 0. In contrast the Raman coupling, formed by a pair of counter propagating laser beams with wavelength λ_R_ = 790 nm, imparted a phase factor exp(−2i*k*_R_*x*). Here, $\hbar k_{\text{R}}=2\pi \hbar /\lambda_{\text{R}}$ is the Raman recoil momentum, giving ϕ/2π≈4/3. We then applied a force by shifting the center of the confining potential along **e**_*x*_, effectively applying a linear potential. Using time-of-flight techniques [27], we measured hybrid momentum/position density distributions *n*(*k*_*x*_, *m*), a function of momentum along **e**_*x*_ and position along **e**_*s*_, as seen in figures 1(c), (d).

## 3. Hall conductivity measurement

**Figure 2.**
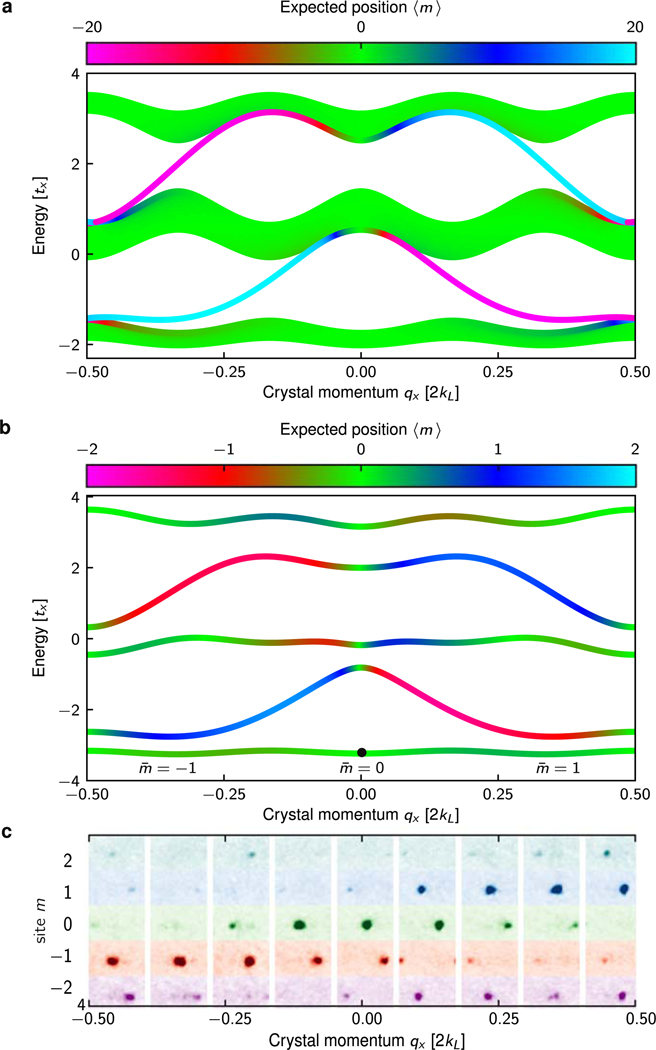
Band structure in an extended system and our 5-site wide ribbon. (a) Band structure of an extended system, with 41 sites along **e**_*s*_, computed for a 4.4*E*_L_ deep 1D lattice (λ_L_ = 1064 nm), 0.5*E*_L_ Raman coupling strength (λ_R_ = 790 nm), and quadratic Zeeman shift *ϵ* = 0*E*_L_, and excluding anisotropy due to Clebsch–Gordan coefficients (see appendix D). The color indicates the expectation value of position along the synthetic direction 〈m〉. (b) Band structure computed using full Hamiltonian for our experimental parameters of 4.4*E*_L_ lattice depth, 0.5*E*_L_ Raman coupling strength, and quadratic Zeeman shift *ϵ* = 0.02*E*_L_, giving $\Phi /\Phi_{0}\approx 4/3,t_{x}=0.154(4)E_{\text{L}},t_{s}=1.97(8)t_{x}$ (see appendix D). The black dot indicates the initial loading parameters. (c) TOF absorption images *n*(*k*_*x*_, *m*) for varying longitudinal crystal momenta *q*_*x*_.

We measured the Hall conductivity beginning with a BEC at *q*_*x*_(*t* = 0) = 0 in the lowest band with transverse modal position ${\bar{m}}_{0}=$ = 0^4^. Figure 2(a) shows the band structure of a system similar to ours, but extended along the **e**_*s*_ direction, with 41 sites. The energy is plotted as a function of crystal momentum along **e**_*x*_, with color indicating the expectation value of position along **e**_*s*_, calculated by diagonalizing the full Hamiltonian with zero quadratic shift and uniform Clebsch–Gordan coefficients (see appendix D). Figure 2(b) shows the band structure of our experimental system, calculated from the full Hamiltonian for our experimental parameters (see appendix D). Note that the lowest, 3rd, and 5th bands of our system are akin to the three bulk bands of the extended system, while the 2nd and 4th bands of our system resemble the edge modes of the extended system.

We applied a force *F*_*x*_ = 0.106(5) *E*_L_/λ_L_ for varying times Δ*t*, directly changing the longitudinal crystal momentum from 0 to a final *q*_*x*_ and giving a transverse Hall displacement from 0 to a final $\bar{m}.$ Figure 2(c) shows a collection of hybrid density distributions, where each column depicts *n*(*k*_*x*_, *m*) for a specific final *q*_*x*_, labeled by the overall horizontal axis. For each column, the change in crystal momentum is marked by the horizontal displacement of the diffraction orders relative to their location in the central *q*_*x*_ = 0 column. The transverse displacement is visible in the overall shift in density along *m* as a function of *q*_*x*_, i.e. between columns.

**Figure 3.**
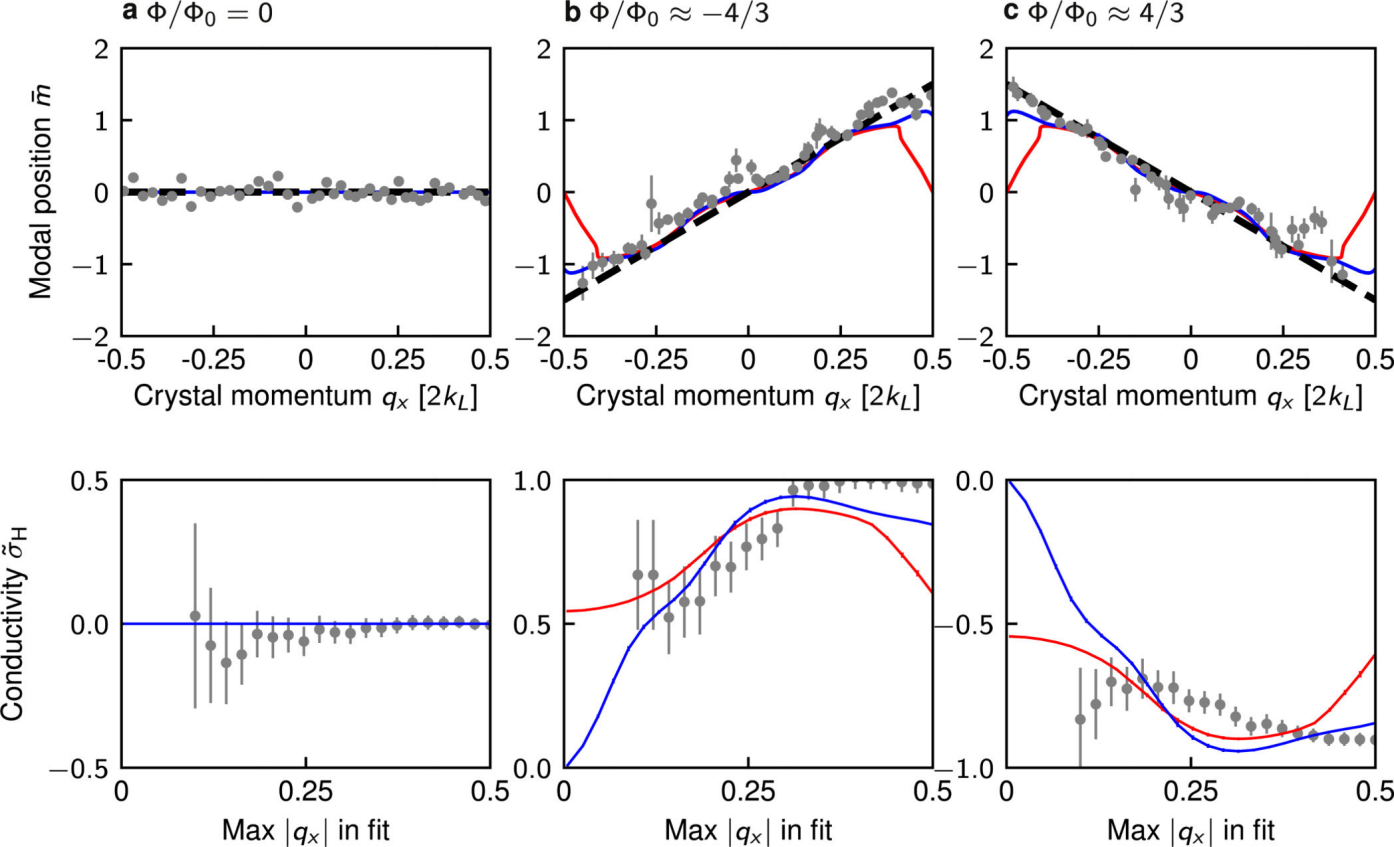
Hall displacement. Top: modal position $\bar{m}$ is plotted as a function of *q*_*x*_ for the 5-site ribbon with flux (a) Φ/Φ_0_ = 0, (b) $\Phi /\Phi_{0}\approx -4/3,$ (c) $\Phi /\Phi_{0}\approx 4/3.$ Gray circles depict the measurements; black dashed lines are the prediction of our simple ${\tilde{\sigma }}_{\text{H}},$ red curves are the expectation from the lowest band theory of our thin ribbon, and blue curves are the result of TDSE calculations for our experimental parameters including force. Bottom: extracted conductivity from the slope of a line of best fit to the data (gray circles), lowest band theory (red lines), and TDSE calculations (blue lines) as a function of maximum $\left|q_{x}\right|$ included in the fit range, for each flux value. As discussed in appendix B, the Φ/Φ_0_ = 0 data was compensated to account for non-adiabaticity in the loading procedure.

Figure 3 (left) quantifies this Hall effect by plotting the modal position $\bar{m}$ as a function of *q*_*x*_ for Φ/Φ_0_ = 0, −4/3, and 4/3. The data is represented by gray dots, with uncertainty bars reflecting the propagated standard uncertainty from averaging six identical runs. For zero flux Φ/Φ_0_ = 0 (figure 3(a)), $\bar{m}$ was independent of *q*_*x*_; in contrast, for non-zero flux $\Phi /\Phi_{0}\approx \pm 4/3$ (figures 3(b), (c)), $\bar{m}$ depends linearly on *q*_*x*_ with non-zero slope. These linear dependencies evoke our earlier discussion of the Hall conductance ${\tilde{\sigma }}_{\text{H}},$ in which we anticipated slopes equal to the Chern number. Linear fits to the data give ${\tilde{\sigma }}_{\text{H}}$ = 0.01(1), 0.87(3), and −0.85(3) for zero, negative and positive flux respectively, showing the expected qualitative behavior. The expected slopes, given by the Chern number, σ_H_ = 0, ±1 are indicated by black dashed lines in figure 3 (left).

The red curves in figure 3 (top) show the expected behavior for our 5-site wide system for adiabatic changes in *q*_*x*_ as calculated from exact diagonalization of the full Hamiltonian (see appendix D), always within the lowest band (figure 2(a)), i.e. Bloch oscillations. This theory predicts a nearly linear slope for small *q*_*x*_ sharply returning to $\bar{m}$ = 0 at the edges of the Brillouin zone. A linear fit to this theory produces ${\tilde{\sigma }}_{\text{H}}\approx 0,0.6,$ and −0.6 for zero, negative and positive flux respectively, far from the Chern number. This discrepancy is resolved by recalling that Bloch oscillations require adiabatic motion. As the ribbon width grows, the band gaps at the edge of the Brillouin zone close (see figure 2(a)), making the Bloch oscillation model inapplicable. The departure of the data from the adiabatic theory at the edges of the Brillouin zone indicates a partial break down of adiabaticity was present in our data. To confirm this, we performed time-dependent Schroedinger equation (TDSE) calculations for our experimental parameters, including the magnitude of the force applied. This is displayed by the blue curves in figure 3. Note that the TDSE curves (blue) lie between the lowest band theory (red) and the large system limit (black dashed lines) at the edges of the Brillouin zone, confirming a partial breakdown of adiabaticity (see appendix C for further detail).

One might suspect that limiting the domain of the linear fit such that band edge effects are excluded would still provide a good measure of the Chern number. However, as shown in figure 3 (bottom), the slope of the best fit line for non-trivial topologies, and thus the measured conductivity, depends highly on the selected domain for both the theoretical (red), the experimental (black) and numerical (blue) data, and the appropriate choice of range is ambiguous. We conclude that for an extremely narrow system such as ours, a conductivity measurement is insufficient for determining the Chern number [20].

## 4. Chern number measurement via TKNN Diophantine equation

**Figure 4.**
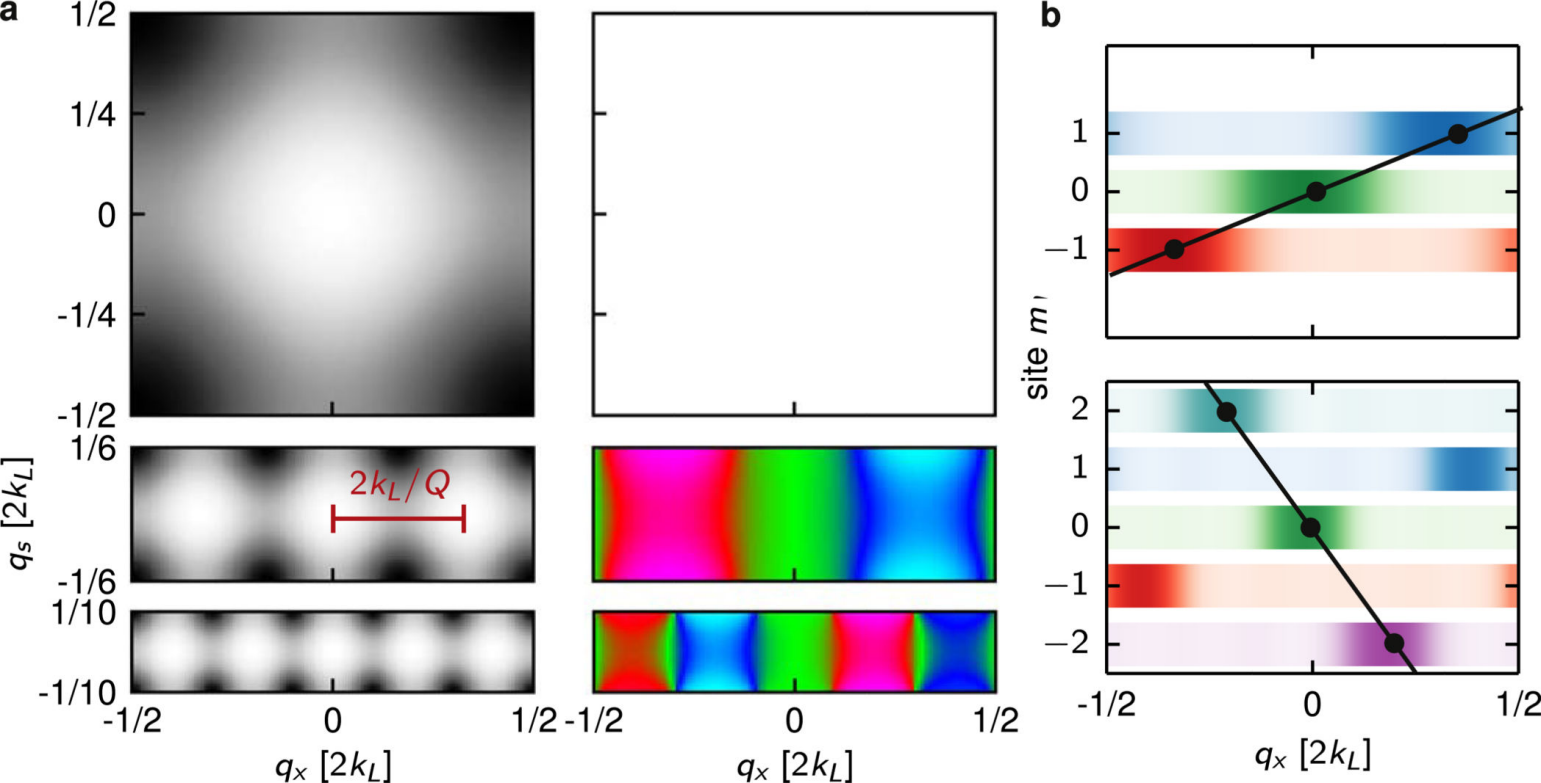
Chern number from the TKNN equation. (a) Lowest band energy (left) and expected position along **e**_*s*_, 〈m〉 (right) within the Brillouin zone in an extended 2D system, where *q*_*x*_ and *q*_*s*_ are crystal momenta along **e**_*x*_ and **e**_*s*_, respectively. Top. Φ/Φ_0_ = 0. Middle. Φ/Φ_0_ = 1/3: Brillouin zone shrinks by a factor of 3 and becomes three-fold degenerate, distance between adjacent energy minima spaced by 2*k*_L_/*Q* is labeled. Each minimum corresponds to a different expected position along **e**_*s*_. Bottom. Φ/Φ_0_ = 2/5. (b) Fractional population in each spin state in the lowest band at *q*_*s*_ = 0. Top. Φ/Φ_0_ = 1/3. Bottom. Φ/Φ_0_ = 2/5. A momentum shift along **e**_*x*_ of 2*k*_L_/*Q* is accompanied by an integer number of spin flips *C*. A line connecting magnetic states separated by 2*k*_L_/*Q*, with slope *C* = 1 (top) and −2 (bottom), is indicated.

To better identify Chern numbers, we relate the TKNN equation (equation (1)) to the physical processes present in our system. Although the Hofstadter Hamiltonian in equation (2) is only invariant under *m*-translations that are integer multiples of *Q*, a so-called ‘magnetic-displacement’ by Δ*m* = 1 along **e**_*s*_ accompanied by a displacement in crystal momentum along **e**_*x*_ by Δ*q*_*x*_/2*k*_R_ = *P/Q* leaves equation (2) unchanged—this is the magnetic translation operator. Together, these symmetry operations give a *Q*-fold reduction of the Brillouin zone along **e**_*s*_, and add a *Q*-fold degeneracy, as illustrated in figure 4(a) for Φ/Φ_0_ = 0, 1/3, and 2/5. Recalling that the Brillouin zone is 2$\hbar k_{\text{L}}$ periodic along **e**_*x*_, it follows that a displacement by 2*k*_L_/*Q* to the nearest symmetry related state involves an integer *C* magnetic displacements, shown in figure 4(b) for Φ/Φ_0_ = 1/3 and 2/5, given by solutions to 2*k*_L_*s* − 2*k*_R_ *C* = 2*k*_L_/*Q*, where s counts the number oftimes the Brillouin zone was ‘wrapped around’ during the *C* vertical displacements. Because this is exactly the TKNN equation (1), we identify *C* as the Chern number. Both *C* and *s* directly relate to physical processes. First, each time the Brillouin zone is wrapped around implying a net change of momentum by 2$\hbar k_{\text{L}}$ a pair of photons must be exchanged between the optical lattice laser beams. Second, each change of *m* by 1 must be accompanied by a 2$\hbar k_{\text{R}}$ recoil kick imparted by the Raman lasers as they change the spin state. This physical motivation of the TKNN equation remains broadly applicable even for our narrow lattice, providing an alternate signature of the Chern number.

**Figure 5.**
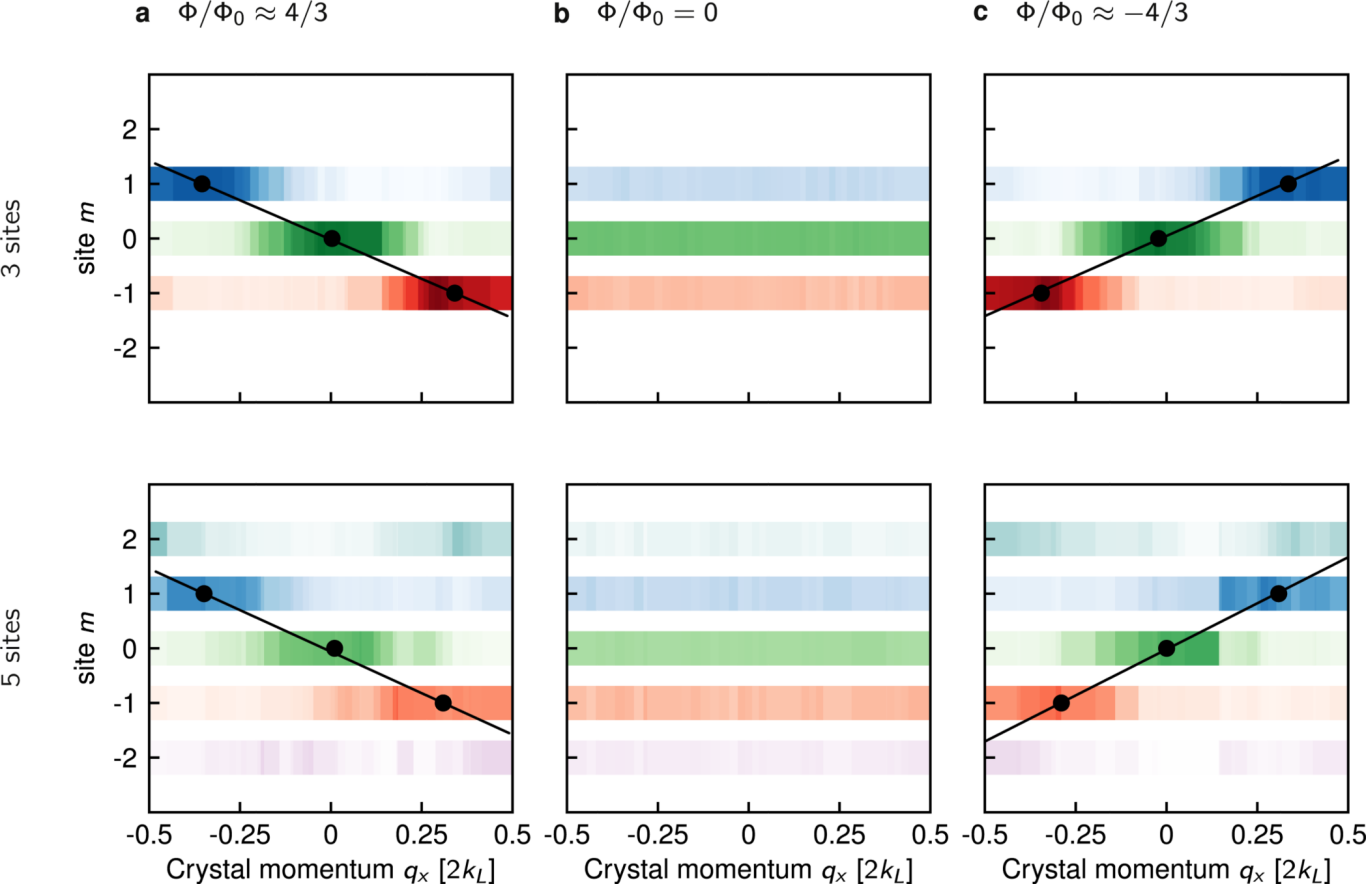
Chern number measurement. Lowest band fractional population measured as a function of crystal momentum in the **e**_*x*_ and position in the **e**_*s*_. Darker color indicates higher fractional population. In the Raman-coupled cases, the points represent the fitted population maxima and the Chern number is extracted from the best fit line to those points. (a) 3-site (left) and 5-site (right) systems with positive flux. (b) 3-site (left) and 5-site (right) system with zero flux. (c) 3-site (left) and 5-site (right) systems with negative flux. The parameters for 3-site data were identical to those for 5-site data, see figure 3(a), except *t*_*s*_ = 0.98(8) *t*_*x*_.

Figure 5 shows the full evolution of fractional population in each *m* site as a function of crystal momentum *q*_*x*_ in the lowest band. The black circles locate the peak of the fractional population in each spin state. We identify the locations of those peaks as the crystal momenta at which the atoms were displaced by a single lattice site along **e**_*s*_ starting at *q*_*x*_ = 0, similar to the suggestions in [19,20]. We associate the Chern number with the slope of a linear fit through the three peak locations. The dependence of Chern number extracted in this way on the strength of applied force is much weaker than the Hall conductivity approach (see appendix C). For the 3-site wide ribbon, we measured a Chern number of0.99(4), −0.98(5) for negative and positive flux respectively^5^, in agreement with the exact theory as calculated from the full Hamiltonian (see appendix D), which predicts ±0.97 (1), with uncertainties reflecting fit uncertainty of peak locations. For the 5-site wide ribbon, we measured 1.11 (2) −0.97(4), close to the theoretical prediction of ±1.07(1).

**Figure 6.**
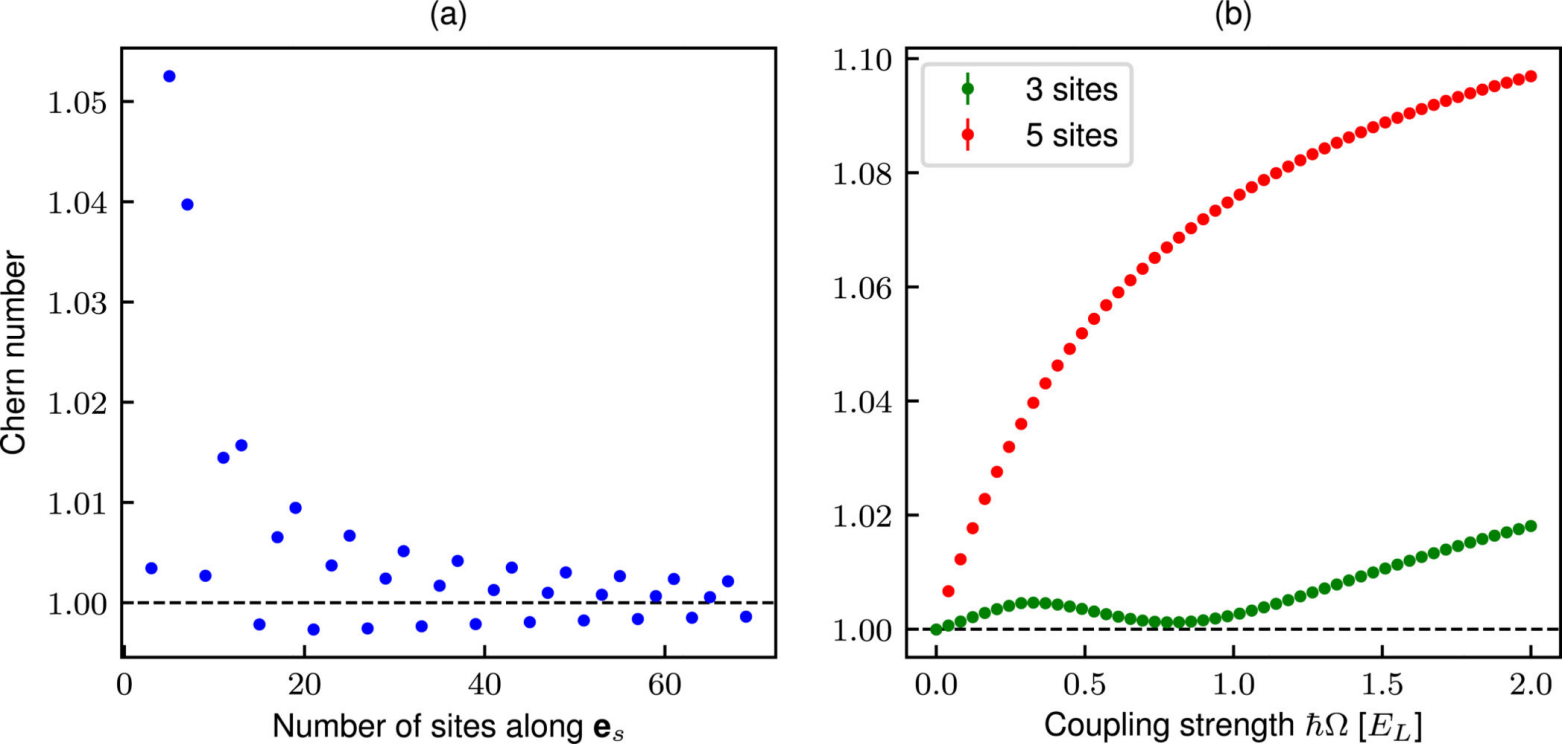
Dependence of Chern number inferred via the TKNN Diophantine equation method on system size and tunneling. (a) Chern number dependence on number of sites along **e**_*s*_. (b) Chern number dependence on coupling strength along the synthetic direction for 3 and 5 site systems.

The deviation from unity results from $\Phi /\Phi_{0}-4/3\approx 0.01,$ a non-zero quadratic Zeeman shift, and *t*_*s*_ > *t*_*x*_ allowing hybridization of states in the vicinity of the edge (see figure 2 in [20]). The dependence of a Chern number inferred with this technique on both the size of the system along **e**_*s*_ and the tunneling *t*_*s*_ is studied in figure 6. Figure 6(a) shows the dependence of the Chern number on the width of the ribbon, from our experimental parameters of 3 and 5 sites to an extended system of 70 sites. As seen in the figure, as the system size grows the measured Chern number converges to the expected value of 1. These were calculated from the full Hamiltonian (appendix D), assuming uniform tunneling along **e**_*s*_, with tunneling *t*_*x*_ = 0.5*E*_L_, synthetic direction coupling $\hbar \Omega =0.5E_{\text{L}}$ and no quadratic shift. Figure 6(b) shows the dependence of the measured Chern number on the coupling strength along the synthetic direction ℏΩ for lattice widths relevant to our experiment—3 and 5 sites. We used the same Hamiltonian and parameter values listed above. In the limit of vanishing tunneling, both the 3 and 5-site wide Chern number converge to the exact integer value of 1. This supports the hypothesis that deviation from unity at non-zero coupling strengths is a consequence of the hybridization of states in the vicinity of the edge, which is facilitated by stronger couplings.

## 5. Conclusion

Our direct microscopic observations of topologically driven transverse transport demonstrate the power of combining momentum and site-resolved position measurements. With the addition of interactions, these systems have been shown to display chiral currents [30], and with many-body interactions are predicted to give rise to complex phase diagrams supporting vortex lattices and charge density waves [31–33]. Realizations of controlled cyclic coupling giving periodic boundary conditions [26] along **e**_*s*_ could elucidate the appearance of edge modes as the coupling between two of the three states is smoothly tuned to zero. In addition, due to the non-trivial topology as well assign the low heating afforded by synthetic dimensional systems, a quantum Fermi gas dressed similarly to our system would be a good candidate for realizing fractional Chern insulators [34].

## Figures and Tables

**Figure C1.**
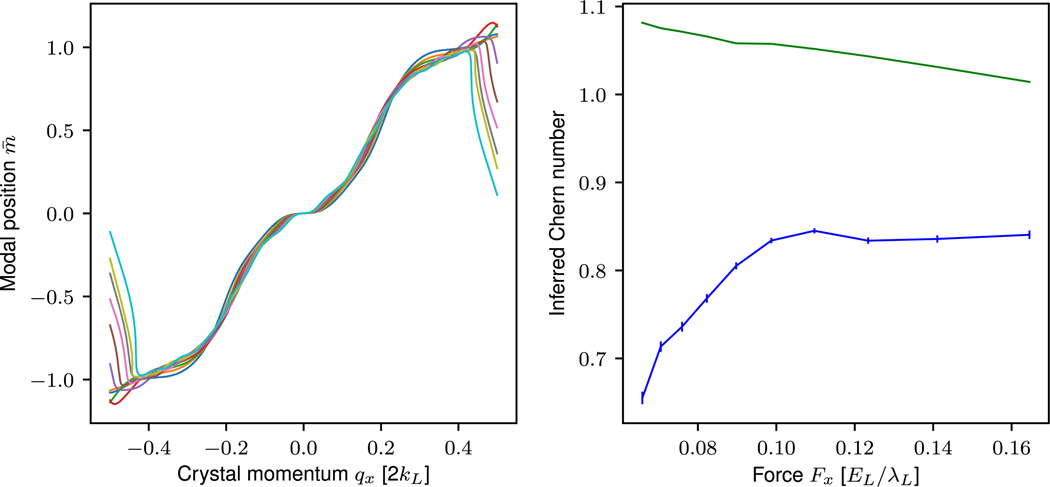
Dependence of Hall conductivity measurement on force in the 5-site strip. Left: modal position $\bar{m}$ as a function of crystal momentum for different magnitudes of applied force, obtained via numerical solutions of the time-dependent Schroedinger equation. The smallest force (cyan) resulted in adiabatic evolution, while the strongest force (red) resulted in almost full non-adiabaticity. Right: measured Chern number as a function of applied force, as inferred from the Hall conductivity (blue) and the TKNN Diophantine equation method (green).

**Figure D1.**
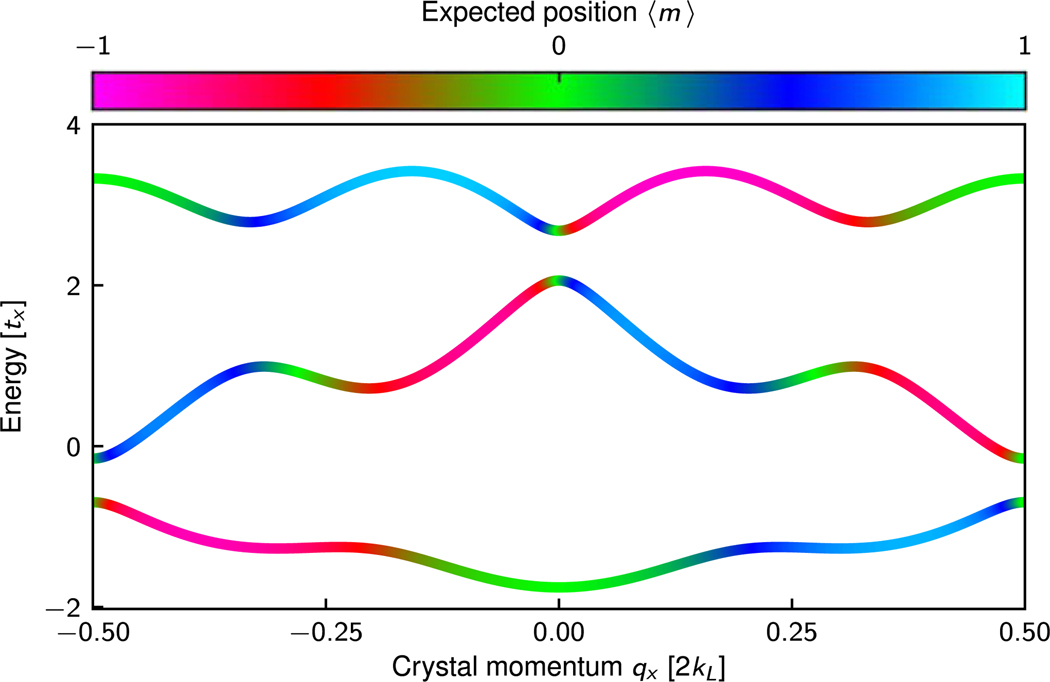
Band structure of the 3-site system for our experimental parameters, with color representing the expectation value of position along **e**_*s*_, 〈m〉.

## Footnotes

3 Subject to the constraint |C|⩽|Q|/2 [16,21]. The integer *s* has no bearing on our argument, but has been interpreted as the charge transported when the periodic potential is adiabatically displaced [22, 23].

4 We define the modal position $\bar{m}$ as the center of a Gaussian fit to the population distribution along **e**_*s*_.

5 Our Chern number extraction scheme fails for the rf case as the fractional populations are flat and there is no peak. We therefore assign a Chern number of 0 to flat distributions.

## Appendix A. Experimental detail

We created nearly pure ^87^Rb BECs in a crossed optical dipole trap [27] with frequencies $\left(\omega_{x},\omega_{y},\omega_{z}\right)/2\pi =(27.1(2),58.4(8),94.2(5))\;\text{Hz}.$ We deliberately used small, low density BECs with $\approx 10^{3}$ atoms to limit unwanted scattering processes in regimes of dynamical instability [35]. At various times in the sequence, we used coherent rf and microwave techniques to prepare the hyperfine $|F,m_{F}\rangle$ state of interest. The 1D optical lattice was always ramped on linearly in 300 ms. For non-zero *ϕ*, we turned on the Raman beams adiabatically in 30 ms after ramping on the lattice. For *ϕ* = 0 we used adiabatic rapid passage starting in *m*_*F*_ = −*F* and swept the bias magnetic field in ≈50 ms to resonance. We applied forces by spatially displacing the optical dipole beam providing longitudinal confinement (by frequency shifting an acousto-optic modulator),

effectively adding a linear contribution to the existent harmonic potential for displacements small compared to the beam waist.

## Appendix B. Rf correction

In experiments where the tunneling along **e**_*s*_ was induced by a uniform rf magnetic field (Φ/Φ_0_ = 0), our loading procedure had remnant non-adiabaticity that led to temporal oscillations in the fractional populations in different *m* states at the 40% level. To separate the effects due to this non-adiabaticity from transverse transport, we performed the experiment with identical preparations without applying the longitudinal force. We then used the observed oscillations as a function of time without an applied force as a baseline, and report the difference in fractional populations between that baseline and the cases where the force was applied.

## Appendix C. Hall conductivity

The current density can be expressed as $j_{⊥}=n_{2\text{D}}v_{⊥}e,$ where *n*_2D_ is the 2D charge carrier density, $v_{⊥}$ is the transverse velocity and *e* is the electron charge. Using $\sigma_{\text{H}}E_{\|}=F_{\|}\sigma_{\text{H}}/e,$ and choosing some increment of time Δ*t*, we have $v_{⊥}=\Delta x_{⊥}/\Delta t,\;\text{and}\;F_{\|}=\hbar \Delta q_{\|}/\Delta t,\;\text{where}\;q_{\|}$ is the crystal momentum along the direction of the force. Re-expressing *n*_2D_ in number of carriers *N* per plaquette, defining $\Delta x_{⊥}$ as transverse displacement in units of lattice periods, we obtain $NG\Delta x_{⊥}/\Delta q_{\|}=\sigma_{\text{H}}R_{\text{K}}.$ We set *N* = 1 as we are looking at motion of single atoms.

The dependence of the measured Hall conductivity for our experimental parameters as a fucntion of the magnitude of applied force is shown in figure C1. The modal position for every point in the Brillouin zone was calculated by numerically solving the TDSE. These are shown in the left panel of the figure, for forces 0.165, 0.141, 0.123, 0.11, 0.099, 0.09, 0.082, 0.076, 0.071 and 0.066 *E*_L_/λ_L_. The right panel shows the measured Hall conductivity from a linear fit to the full range of crystal momenta as a function of applied force (blue line). For comparison, we also show the Chern number as inferred via the TKNN Diophantine equation method as a function ofapplied force (green line).

## Appendix D. Full Hamiltonian

The spin dependent Hamiltonian, in the presence of Raman coupling and a 1D optical lattice can be written as

$$H=\sum_{m=-F,n=-\infty }^{F,\infty }H_{0}+H_{\text{R}}+H_{\text{L}},$$

**Table D1.** Summary of experimental parameters.

Parameter	Value
*V*	4.4(1) *E*_L_
*t_x_*	0.154(4) *E*_L_
*t_s_*	3-site Raman: 0.97(8) *t*_*x*_; 5-site Raman: 1.97(8) *t*_*x*_; rf: 7.4(5) *t_x_*
*ϵ*	0.02 *E*_L_
*δ*	0.00(2) *E*_L_
*F_x_*	0.106(5) *E*_L_/λ_L_

where the diagonal term

$$H_{0}=\left(\hbar^{2}{\left(q_{x}-2m\Phi /\Phi_{0}-2n\right)}^{2}k_{\text{L}}^{2}/2m_{\text{Rb}}+h\delta m+\epsilon m^{2}\right)\;\;\times |q_{x}+n2k_{\text{L}},m\rangle \;\langle q_{x}+n2k_{\text{L}},m|$$

includes the kinetic energy as well as the two-photon Raman detuning from resonance *δ* and the quadratic Zeeman shift *ϵ*. Here, *m* represents atomic spin, *n* represents lattice order in the momentum basis, *q* is the longitudinal crystal momentum, and *m*_Rb_ is the atomic mass. The second term represents the Raman coupling with coupling strength Ω, with anisotropic tunneling arising from the spin-dependent prefactor (Clebsch–Gordan coefficient):

$$H_{\text{R}}=\hbar \Omega \sqrt{F(F+1)-m(m+1)}/2\sqrt{2}|q_{x}+n2k_{\text{L}},m\rangle \;\langle q_{x}+n2k_{\text{L}},m+1|+\text{h}.\text{c}.$$

Here, h.c. stands for Hermitian conjugate. The third term represents lattice coupling to higher order lattice states, with lattice depth *U*:

$$H_{\text{L}}=U/4|q_{x}+n2k_{\text{L}},m\rangle \;\langle q_{x}+(n+1)2k_{\text{L}},m|+\text{h}.\text{c}.$$

In our experiment, $\hbar \Omega =0.5(2)E_{\text{L}}$ for Raman coupling and $\hbar \Omega =0.57(1)E_{\text{L}}$ for rf coupling, *U* = 4.4(4)*E*_L_ for all preparations. In our calculations, we found that restricting −7 < *n* < 7 was sufficient at our energies.

We choose the appropriate tunneling coefficients *t*_*x*_ and *t*_*s*_ for the approximate tight-binding Hamiltonian equation (2) as those that optimally reproduce the lowest two bands, relevant for our experiment. A summary of our experimental parameters is presented in table D1. The band structure of our 3-site wide ribbon is presented in figure D1, with three bands plotted as a function of crystal momentum *q*_*x*_ colored in accordance with the expected position along **e**_*s*_, 〈m〉.
